# *Lutzomyia longipalpis* Saliva or Salivary Protein LJM19 Protects against *Leishmania braziliensis* and the Saliva of Its Vector, *Lutzomyia intermedia*

**DOI:** 10.1371/journal.pntd.0001169

**Published:** 2011-05-31

**Authors:** Natalia M. Tavares, Robson A. Silva, Dirceu J. Costa, Maiana A. Pitombo, Kiyoshi F. Fukutani, José C. Miranda, Jesus G. Valenzuela, Aldina Barral, Camila I. de Oliveira, Manoel Barral-Netto, Claudia Brodskyn

**Affiliations:** 1 Centro de Pesquisa Gonçalo Moniz, FIOCRUZ, Salvador, Bahia, Brazil; 2 Universidade Federal da Bahia, Instituto Multidisciplinar em Saúde, Campus Anisio Teixeira, Vitoria da Conquista, Bahia, Brazil; 3 Vector Molecular Biology Unit, Laboratory of Malaria and Vector Research, National Institute of Allergy and Infectious Disease, National Institutes of Health, Rockville, Maryland, United States of America; 4 Instituto de Investigação em Imunologia, São Paulo, São Paulo, Brazil; Johns Hopkins Bloomberg School of Public Health, United States of America

## Abstract

**Background:**

*Leishmania* transmission occurs in the presence of insect saliva. Immunity to *Phlebotomus papatasi* or *Lutzomyia longipalpis* saliva or salivary components confers protection against an infection by *Leishmania* in the presence of the homologous saliva. However, immunization with *Lutzomyia intermedia* saliva did not protect mice against *Leishmania braziliensis* plus *Lu. intermedia* saliva. In the present study, we have studied whether the immunization with *Lu. longipalpis* saliva or a DNA plasmid coding for LJM19 salivary protein would be protective against *L. braziliensis* infection in the presence of *Lu. intermedia* saliva, the natural vector for *L. braziliensis*.

**Methodology/Principal Findings:**

Immunization with *Lu. longipalpis* saliva or with LJM19 DNA plasmid induced a Delayed-Type Hypersensitivity (DTH) response against *Lu. longipalpis* as well as against a *Lu. intermedia* saliva challenge. Immunized and unimmunized control hamsters were then intradermally infected in the ears with *L. braziliensis* in the presence of *Lu. longipalpis* or *Lu. intermedia* saliva. Animals immunized with *Lu. longipalpis* saliva exhibited smaller lesion sizes as well as reduced disease burdens both at lesion site and in the draining lymph nodes. These alterations were associated with a significant decrease in the expression levels of IL-10 and TGF-β. Animals immunized with LJM19 DNA plasmid presented similar findings in protection and immune response and additionally increased IFN-γ expression.

**Conclusions/Significance:**

Immunization with *Lu. longipalpis* saliva or with a DNA plasmid coding LJM19 salivary protein induced protection in hamsters challenged with *L. braziliensis* plus *Lu. intermedia* saliva. These findings point out an important role of immune response against saliva components, suggesting the possibility to develop a vaccine using a single component of *Lu. longipalpis* saliva to generate protection against different species of *Leishmania*, even those transmitted by a different vector.

## Author Summary

Leishmaniasis, caused by parasitic protozoa *Leishmania*, is transmitted by bites of female sand flies that, during blood-feeding, inject humans with parasites and saliva. Sand fly saliva has been investigated as a potential vaccine candidate. It was previously shown that immunization with *Lutzomyia longipalpis* saliva or salivary proteins protects against cutaneous and visceral leishmaniasis. In the present study, we evaluated if immunization with *Lu. longipalpis* saliva or DNA plasmid coding for a specific sand fly salivary protein (LJM19) can protect hamsters against *L. braziliensis* plus another sand fly saliva. Immunization with saliva or LJM19 DNA plasmid induced a mononuclear cell infiltrate which can be a marker of protection. The immune response induced by immunization with these insect molecules was able to protect animals against *L. braziliensis* infection as shown by the significant reduction in lesion size, parasite load in the ear and draining lymph node. These data show the important role of immune response against sand fly saliva components, suggesting the possibility to develop vaccines using a single component of saliva against *Leishmania* transmitted by different vectors.

## Introduction

Transmitted by sand flies, the parasitic protozoa of the genus *Leishmania* are the etiological agents of tegumentar or visceral diseases in humans. Differences in *Leishmania* species and the genetic background or immunological status of the host underlie the diverse clinical manifestations in leishmaniasis. *Leishmania braziliensis* is the etiologic agent of American Cutaneous Leishmaniasis (ACL), which is characterized by its chronicity and the possibility to metastasize leading to the muco-cutaneous clinical form [1].

Saliva from sand flies and other blood feeders contains potent pharmacologic components that facilitate blood meal acquisition and evasion of host inflammatory and immune response [2], [3]. Arthropod vector saliva also plays an important role in pathogen transmission. A small amount of vector saliva can intensify parasite or virus infectivity [4]–[6]. On the other hand, immune response to arthropod saliva or to insect bites precludes establishment of the pathogen in the vertebrate host. Recent reports have shown the importance of salivary proteins from sand fly vectors as potential targets for vaccine development against *Leishmania* infection [4], [7]–[9].

Valenzuela *et al* showed that immunization with DNA plasmid coding for *Phlebotomus papatasi* SP15 protein was effective in providing protection against co-inoculation of *L. major* plus Salivary Gland Sonicate (SGS) in mice [8]. Our group also demonstrated that hamsters immunized with DNA plasmid coding LJM19 *Lu. longipalpis* protein were protected against a challenge composed by *L. infantum chagasi* plus *Lu. longipalpis* SGS [9]. These two studies, as proposed before [10], suggest that a Delayed-Type Hypersensitivity (DTH) response is the mechanism underlying the protective anti-*Leishmania* immunity.

Exposure to sand fly saliva does not always result in a protective outcome. Mice immunized with *Lu. intermedia* SGS developed larger lesions and increase in parasite load when challenged with *L. braziliensis* and *Lu. intermedia* SGS [5]. These data suggests that immune responses against components present in saliva from different vectors could alter the outcome of infection, leading to protection or exacerbation of disease. Therefore, we hypothesized that immunization with *Lu. longipalpis* SGS or DNA plasmid coding for LJM19 protein, one of the most abundant *Lu. longipalpis* salivary proteins [11], could revert the non protective effect of *Lu. intermedia* SGS.

## Methods

### Sand flies and salivary gland lysates

*Lutzomyia intermedia*, Corte de Pedra strain, and *Lutzomyia longipalpis*, Cavunge strain, were reared at Centro de Pesquisas Gonçalo Moniz – FIOCRUZ, as described elsewhere [12]. Female sand flies from 5- to 7-day-old were dissected, the salivary glands were removed and transferred to 10 or 20 µl Hepes, 10 mM pH 7.0 NaCl 0.15 M in 1.5 mL polypropilene vials, usually in groups of 20 pairs of glands in 20 µl of Hepes saline, or individually in 10 µl of Hepes saline. Salivary glands were kept at −75 °C until needed. Salivary glands were disrupted by sonication using a Branson Sonifier 450 homogenizer (Branson, Danbury, CT). Salivary homogenates (1 pair of salivary gland per 1 µL) were centrifuged at 10,000 g for 2 min; the supernatants were used in the experiments (SGS).

### DNA plasmid and immunization

DNA plasmid coding for *Lu. longipalpis* salivary protein LJM19 was cloned into the VR2001-TOPO vector and purified as described [13]. Male Golden Syrian Hamsters (*Mesocricetus auratus*) at 10–12 weeks old were obtained from Centro de Pesquisa Gonçalo Moniz Institute FIOCRUZ facility. Groups of hamsters were immunized or not (20 µL) intradermally (i.d.) in the right ear with *Lu. longipalpis* SGS (5 experiments repeated with 3 hamsters per group per time point) equivalent to 0.5 pair of salivary glands (∼1 µg of protein measured by Bradford method [14]) or 20 µg of DNA plasmid (3 experiments repeated with 5 hamsters per group per time point) for three times at 14 day intervals.

### Analysis of the inflammatory immune response in the ear dermis

Following three i.d. injections with saline (8 hamsters per group total), *Lu. longipalpis* SGS (9 hamsters per group total) or LJM19 DNA plasmid (5 hamsters per group total) in the right ear, hamsters were challenged in the left ear dermis with 0.5 pair of *Lu. longipalpis* SGS, that corresponds to approximately 1 µg of protein, or 1 pair of *Lu. intermedia* SGS, which protein concentration is the same. We used different amount of *Lu. longipalpis* and *Lu. intermedia* saliva in order to allow the same concentration of protein from both SGS. Forty-eight hours post challenge, left sample ears were removed and fixed in 10% formaldehyde. Following fixation, tissues were processed, embedded in paraffin and 5 µm sections were stained with hematoxylin and eosin (H&E) and analyzed by light microscopy.

### Leishmania parasites

*L. braziliensis* (strain MHOM/BR/01/BA788) promastigotes were cultivated in Schneider's Insect Medium (Sigma Chemical Co., St Louis, Mo, USA) supplemented with 20% heat-inactivated fetal bovine serum (Gibco, USA), L-glutamine (2 mM), penicillin (100 U/ml), streptomycin (100 µg/ml) at 23 °C for 5–7 days when the parasites reached the stationary-phase.

### Experimental infection

Two weeks after the last immunization, hamsters were challenged i.d. in the left ear with 20 µL of 10^5^ stationary phase promastigotes in the presence of either (a) *L. braziliensis* plus the equivalent to 0.5 pair of salivary gland of *Lu. longipalpis* SGS (∼1 µg of protein) or in the presence of (b) *L. braziliensis* plus one pair of salivary gland-equivalent of *Lu. intermedia* SGS (∼1 µg of protein). Control animals received saline and the same challenge that immunized hamsters (10^5^ stationary phase promastigotes in the presence of either *Lu. longipalpis* or *Lu. intermedia* SGS). Lesion size was measured weekly using a digital caliper (Thomas Scientific, USA). All procedures involving animal experimentation were conducted according to relevant Brazilian guidelines and approved by the Ethical Committee in Animal Use of Centro de Pesquisa Gonçalo Moniz in 2006/03/03, under the number 083.

### Limiting Dilution Assay (LDA)

Parasite load was determined 3, 5 and 8 weeks post-infection using the quantitative Limiting Dilution Assay (LDA) as described by Titus *et al* [15]. Briefly, infected ears and retromaxillar draining lymph nodes were aseptically removed from individual hamster. Tissues were homogenized and diluted in Schneider's Insect Medium (Sigma, St. Louis, MO) supplemented with 10% heat inactivated fetal bovine serum (Gibco, USA), 100 U of penicillin/ml and 100 µg/ml of streptomycin. Homogenate samples were serially diluted into 96-wells plates containing biphasic blood agar (Novy-Nicolle-McNeal) medium and incubated for one week at 23°C. Wells with positive growth were noted at specific dilutions and applying the ELIDA 1986 software to calculate the parasites burdens in the tissues.

### RNA isolation and quantitative Real-Time PCR

Total RNA was extracted from the draining lymph nodes using Trizol reagent (Invitrogen, USA) 3, 5 and 8 weeks after infection. First-strand cDNA synthesis was performed with 1–2 µg of RNA in a total volume of 25 µL by using SuperScript II (Gibco, Carlsbad, CA, USA). DNA was amplified in the thermocycler (Mastercycler gradient – Eppendorf, USA) with an initial pre-incubation at 72°C for 5 minutes, followed by amplification of the target DNA at 42°C for 50 minutes. A standard curve was generated for each set of primers and efficiency of each reaction was determined. The expression levels of genes were normalized to GAPDH levels. The results are expressed in fold change over control. Oligonucleotide primers used were: GAPDH (reverse 5′-CTGACATGCCGCCCTGGAG-3′ and forward 3′-TCAGTGTAGCCCAGGATGCC-5′); IFN-γ (reverse 5′-GAAGCTCACCAAGATTCCGGTAA-3′ and forward 3′-TTTTCGTGACAGGTGAGGCAT-5′); IL-10 (reverse 5′-AGACGCCTTTCTCTTGGAGCTTAT-3′ and forward 3′-GGCAACTGCAGCGCTGTC-5′); and TGF-β (reverse 5′-GCTACCACGCCAACTTCTGTC-3′ and forward 3′-TGTTGGTAGAGGGCAAGG-5′).

### Statistical analysis

The experiments with total saliva were repeated five times with three hamsters per groups per time point, whereas experiments using LJM19 DNA plasmid were repeated three times with five hamsters per group per time point. Results were expressed as mean ± standard error of the mean or median with interquartile range. Comparisons among immunized and non-immunized control groups were done by Mann-Whitney non-parametric t test, in the following situations: 1. unimmunized and challenged with *Lu. longipalpis* SGS + *L. braziliensis* X immunized with *Lu. longipalpis* SGS and challenged with *Lu. longipalpis* SGS + *L. braziliensis*; 2. unimmunized and challenged with *Lu. intermedia* SGS + *L. braziliensis* X immunized with *Lu. longipalpis* SGS and challenged with *Lu. intermedia* SGS + *L. braziliensis.* In the experiments using hamsters immunized with LJM19 DNA plasmid, we employed the same statistical analysis, but the control group consisted of animals injected with empty plasmid. One-way ANOVA (Kruskal-Wallis) analysis with Dunn's post-test was done to compare different groups non-immunized or immunized with SGS and LJM19 DNA plasmid (Figure S2). All the statistical analysis was done using Graph Pad 5.0 software program. The course of disease was plotted individually and the area under each resulting curve (AUC) was calculated using Graph Pad 5.0 software program. The results were considered statistically significant when p<0.05.

## Results

### Immunization with *Lu. longipalpis* SGS or LJM19 salivary protein induces cellular response in hamsters

**Figure 1:**
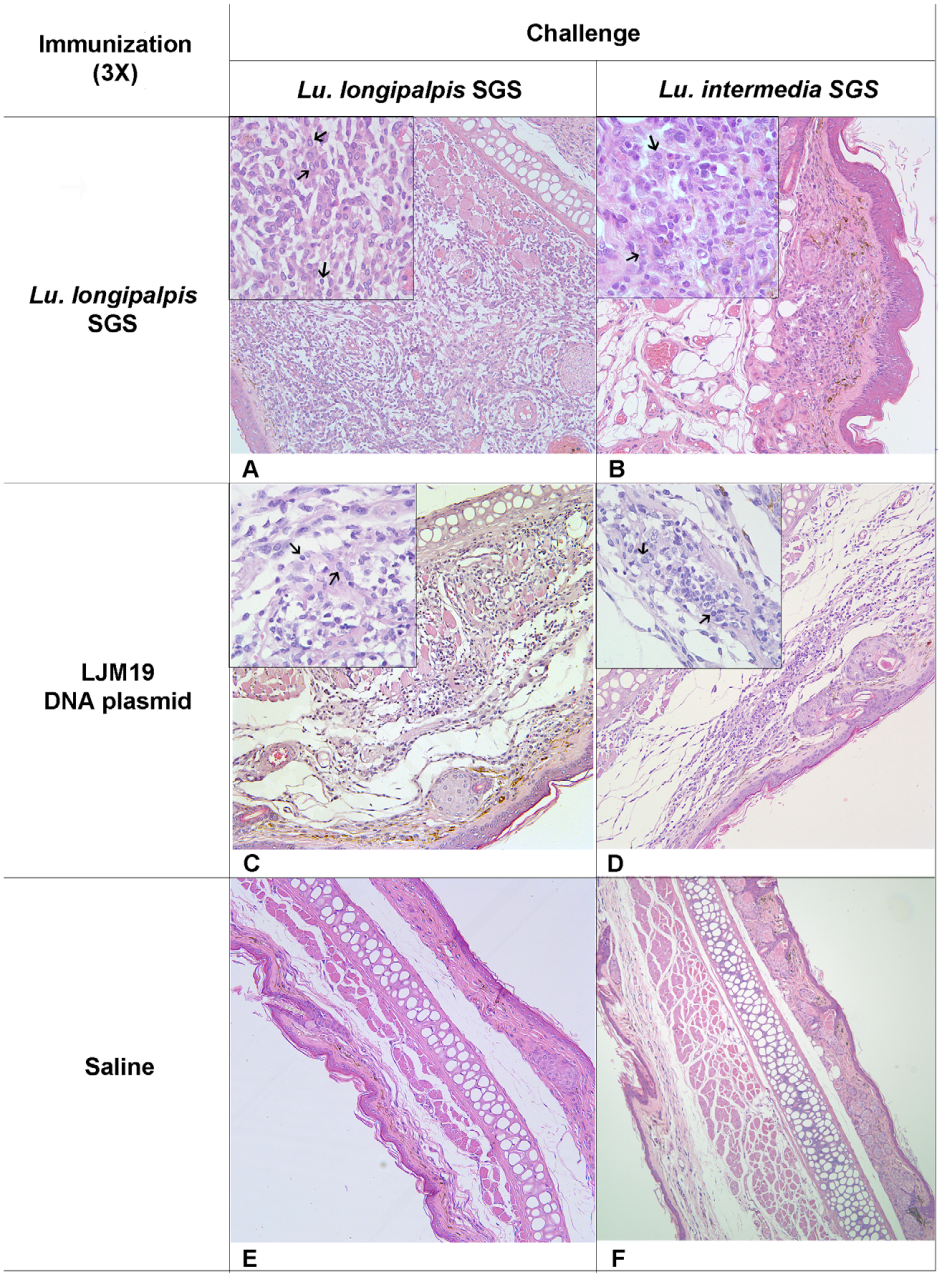
Cellular response against sand fly saliva in hamsters. Hamsters were immunized three times with *Lu. longipalpis* SGS (A–B; 9 hamsters per group total), LJM19 DNA plasmid (C–D; 5 hamsters per group total) or were inoculated with saline (E–F; 8 hamsters per group total) in the right ear. After the last immunization, hamsters were challenged in the left ear dermis with *Lu. longipalpis* or with *Lu. intermedia* SGS. The cellular infiltration was observed by light microscopy stained using H&E at 100x (A–F) and the left corner inserts at 1000x with arrows indicating mononuclear cells (A–D). Experiments were repeated three times and the photographs are representative from each group.

DTH response against salivary proteins can be a marker or correlate of protection against a challenge of parasite plus saliva [8], [9]. To evaluate the induction of DTH reaction by immunization with *Lu. longipalpis* SGS or by LJM19 DNA plasmid, immunized hamsters were challenged in the contralateral ear using either *Lu. longipalpis* or *Lu. intermedia* SGS. Immunization and challenge with *Lu. longipalpis* SGS induced an intense mononuclear cell infiltrate and edema, whereas the challenge with *Lu. intermedia* SGS elicited a smaller recruitment of mononuclear cells and edema (Fig. 1A). Hamsters immunized with LJM19 DNA plasmid and challenged with *Lu. longipalpis* or *Lu. intermedia* SGS also resulted in a DTH response, but with less recruitment of mononuclear cells and more pronounced edema. Control hamsters injected with saline and challenged with SGS exhibited no edema and scarce inflammatory cells (Fig. 1E,F). The observed inflammatory response in all immunized groups consisted predominantly of mononuclear cells (inserts in Fig. 1A–D), characteristic of a typical DTH response.

Immunization with *Lu. longipalpis* SGS induced anti-saliva antibodies as assayed by ELISA. LJM19 immunization did not elicit antibodies to saliva as previously shown [9] (Fig. S1).

### The outcome of immunization with *Lu. longipalpis* SGS followed by infection with *L. braziliensis*

**Figure 2:**
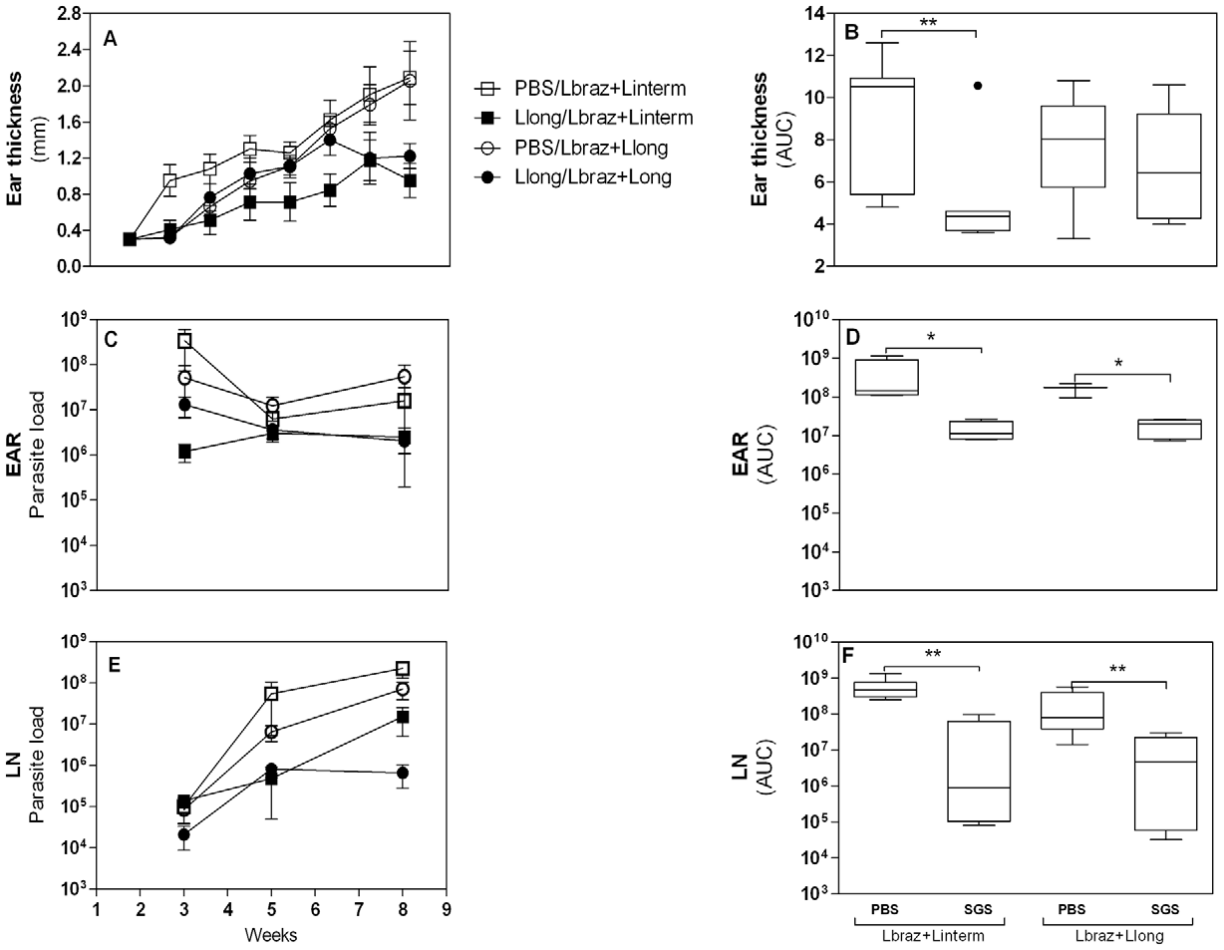
Ear thickness and parasite load in *Lu. longipalpis* SGS immunized hamsters following infection with *L. braziliensis*. Hamsters were inoculated three times in the right ear dermis with *Lu. longipalpis* SGS (closed symbols) or with saline (open symbols) and were infected in the left ear with 10^5^ *L. braziliensis* stationary promastigotes in the presence of *Lu. intermedia* SGS (squares; 7 hamsters per group total) or *Lu. longipalpis* SGS (circles, 8 hamsters per group total). The course of lesion development was monitored weekly (A). The points represent the means and the vertical bars represent standard errors of the means. The areas contained under the curves (AUC) obtained in A for each group was compared (B). The parasite number in the ear (C, D) or draining lymph node (E, F) was evaluated by Limiting Dilution Assay (LDA), estimated by ELIDA in 3, 5 and 8 weeks after the infection. Each bar represents the mean and the vertical bars represent standard errors of the means. The disease burden of the ear (C) or draining lymph node (E) show reduced parasite load in immunized hamsters evaluated by Mann-Whitney non-parametric t test. The areas contained under the curves (AUC) obtained in C and E for each group was compared (D, F) and the experiments were repeated five times. *p<0.05; **p<0.01.

The onset of lesion development in naive hosts was at two weeks post-infection and lesions progressed until 12 weeks (data not shown), confirming the high susceptibility of hamsters to *Leishmania* infection [9], [16], [17]. After this period, the animals were euthanized due to the lesions severity. Disease burden was calculated by weekly measure of ear thickness and by comparison of the area under the resulting curves, as explained in statistical analysis section in Material and Methods. Immunization with *Lu. longipalpis* SGS resulted in significantly (p = 0.011) smaller ear thickness following challenge with parasites plus *Lu. intermedia* SGS as compared to the control group (Fig. 2A–B). The mean of ear thickness ranged between 0.5 mm and 1.0 mm in immunized hamsters during the infection compared with the range of 1.0 mm to 2.0 mm in the control group (Fig. 2A). Parasite load in the ear (from 1.4×10^8^ in unimmunized group to 1.1×10^7^ in immunized group, p = 0.029) and in the lymph node (from 4.6×10^8^ to 9.0×10^5^, p = 0.001) was also significantly reduced in this group (Fig. 2C–F). No significant differences in ear thickness were observed in the group challenged with parasites plus *Lu. longipalpis* SGS (Fig 2A–B). However, there was a significant reduction in ear (from 1.7×10^8^ to 2×10^7^, p = 0.036) and lymph node (from 8×10^7^ to 4.6×10^6^, p = 0.009) parasite load in this group (Fig. 2C–F).

### Reduction of IL-10 and TGF-β is involved in the protection against *L. braziliensis* infection in hamsters immunized with *Lu. longipalpis* SGS

**Figure 3:**
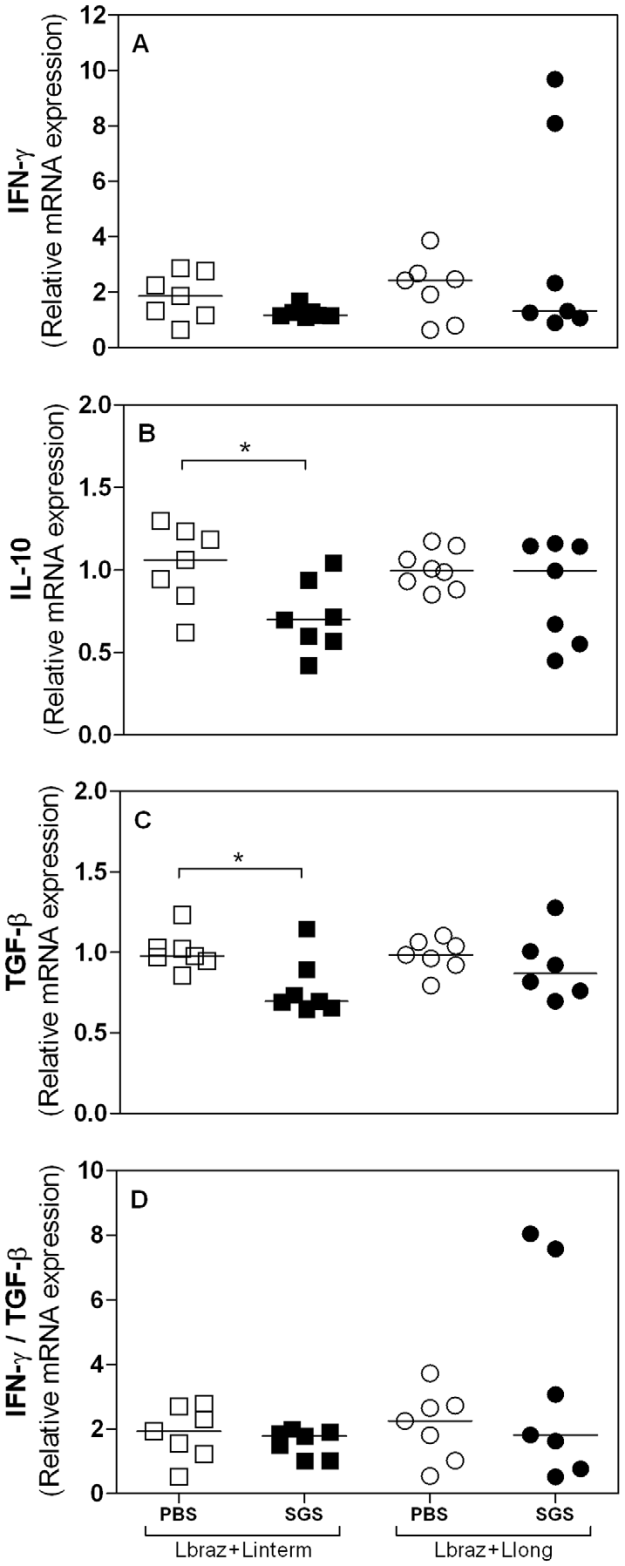
Cytokine expression in lymph node cells from hamsters immunized with *Lu. longipalpis* followed by *L. braziliensis* infection. Hamsters (7 per group total) were inoculated three times in the right ear with *Lu. longipalpis* SGS (closed symbols) or with saline (open symbols) and were challenged in the left ear with 10^5^ *L. braziliensis* in the presence of *Lu. intermedia* (squares) or *Lu. longipalpis* SGS (circles). The IFN-γ (A), IL-10 (B) and TGF-β (C) relative mRNA expression was evaluated by Real-Time PCR, 5 weeks after the infection. Points represent each animal, experiments were repeated five times and were evaluated by Mann-Whitney non-parametric t test. *p<0.05; **<0.01.

We studied the cytokine profile of immunized and infected hamsters three, five and eight weeks post challenge by real time PCR. At five weeks post-infection, IFN-γ levels were similar among all groups (Fig. 3A). Similar results were observed at all time points studied (Fig. S2A and D). There was a significant reduction in IL-10 expression (p = 0.026) in hamsters immunized with *Lu. longipalpis* SGS and challenged with *L. braziliensis* plus *Lu. intermedia* SGS (Fig. 3B) compared to control unimmunized group. A significant reduction was also observed at three (p = 0.021) and eight (p = 0.043) weeks post infection (Fig. S2B and E). We also observed a significant reduction in the expression of TGF-β (p = 0.030) in hamsters immunized with *Lu. longipalpis* SGS and challenged with *L. braziliensis + Lu. intermedia* SGS as compared to unimmunized control group (Fig. 3C). Similar results were obtained at 3 weeks post-infection (p = 0.040, Fig. S2C). However, the group of hamsters immunized with *Lu. longipalpis* SGS and challenged with *L. braziliensis* plus *Lu. longipalpis* SGS showed significant reduction in TGF-β expression only at three weeks after infection (p = 0.050, Fig. S2C). We also analyzed the ratio IFN-γ/TGF-β and there were not significant differences between the immunized and non-immunized groups (data not shown).

### The influence of immunization with DNA plasmid coding LJM19 protein from *Lu. longipalpis* sand fly in hamsters infected with *L. braziliensis*

**Figure 4:**
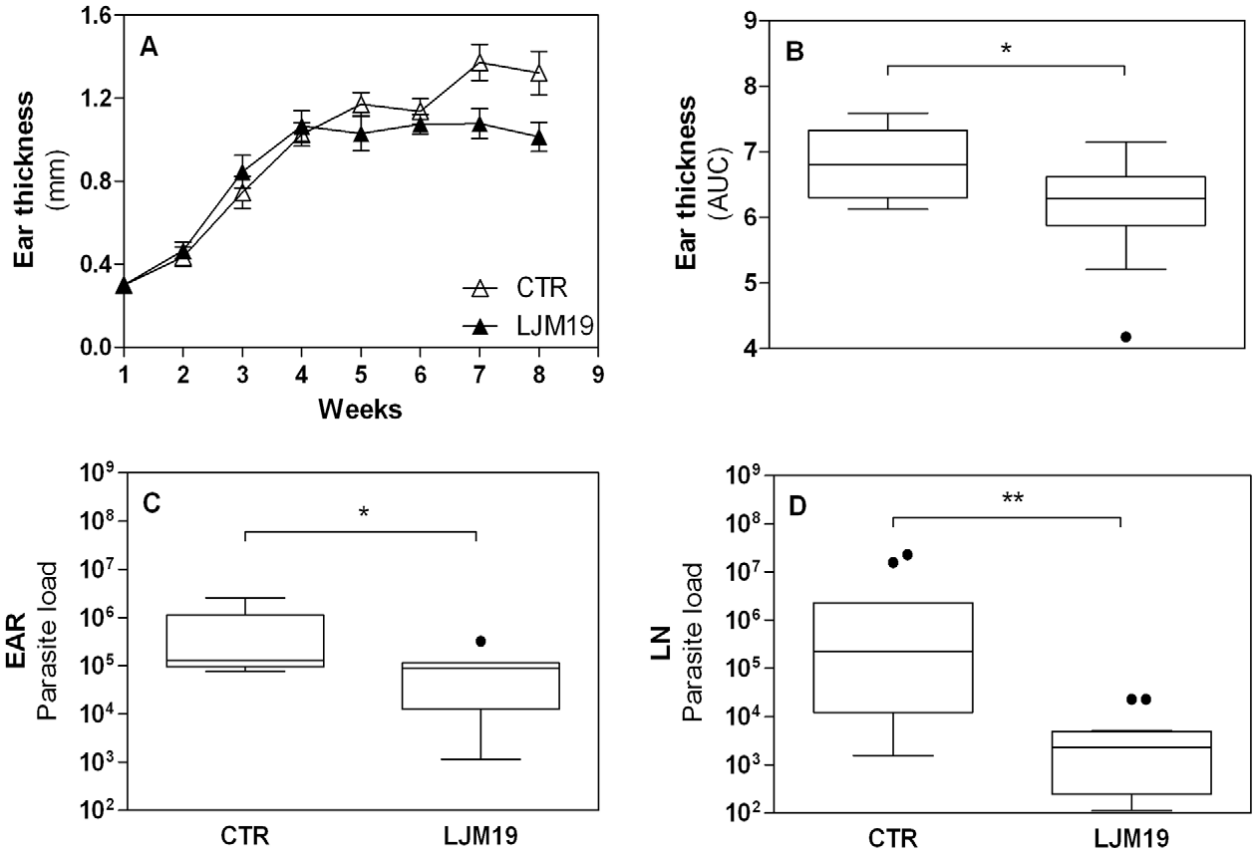
Ear thickness and parasite load in LJM19 immunized hamsters following infection with *L. braziliensis*. Hamsters (12 per group total) were inoculated three times in the right ear with DNA plasmid coding LJM19 salivary protein (closed triangle) or empty DNA plasmid (CTR - open triangle) and were challenged intradermally in the left ear with 10^5^ *L. braziliensis* stationary promastigotes in the presence of *Lu. intermedia* SGS. The course of lesion development was monitored weekly and points represent the means and standard errors of the means (A). The areas contained underneath the curves (AUC) obtained in A for each group was compared (B). Five weeks after the infection, the parasite load was evaluated in the ear (C) and draining lymph node (D) by LDA, estimated by ELIDA. Experiments were repeated three times and were evaluated by Mann-Whitney non-parametric t test. *p<0.05; **p<0.01.

We recently showed that immunization with a DNA plasmid coding for the salivary protein LJM19, one of the most abundant secreted proteins in *Lu. longipalpis* saliva, protected hamsters against visceral leishmaniasis [9]. Since *Lu. longipalpis* SGS protect hamsters against an infection with *L. braziliensis* (Fig. 2), we tested if immunizations with LJM19 DNA plasmid could also protect against *L. braziliensis* infection. This hypothesis is based on the fact that immunization with LJM19 DNA plasmid also induced a DTH response against *Lu. intermedia* SGS (Fig. 1D). Hamsters immunized with LJM19 DNA plasmid were challenged with *L. braziliensis* + *Lu. intermedia* SGS. As shown in Fig. 4A, the onset of lesion development is at three weeks post infection in both groups: LJM19 DNA plasmid immunized and empty DNA plasmid control. At four weeks, the ear thickness in immunized hamsters peaked around 1.0 mm and is maintained without alterations. In control group, the ear thickness increased up to 1.5 mm until eight weeks post infection (Fig. 4A). Importantly, a significant reduction (p = 0.038) in disease burden was observed in immunized compared to control group (Fig. 4B).

At the same time point, we also evaluated the control of *L. braziliensis* infection measuring the parasite load of infected ears (Fig. 4C) and in draining lymph nodes (Fig. 4D). The results showed a significant reduction in parasite load in LJM19 DNA plasmid immunized group (8.9×10^4^ in the ear and 2.3×10^3^ in the lymph nodes) compared with empty DNA plasmid control group (1.3×10^5^ in the ears, p = 0.05 and 2.3×10^5^ in the lymph nodes p = 0.032). Similar results in parasite load of the dLN were obtained at 3 weeks post-infection (Fig. S3). However, at 8 weeks post-infection there is no difference in parasite load between immunized and control groups (data not shown). Interestingly, the protection induced by immunization with *Lu. longipapis* SGS or LJM19 DNA plasmid was similar and no significant differences were observed between them (Fig. S4).

### Increased IFN-γ/TGF-β ratio correlates with the protection of LJM19 immunized hamsters against *L. braziliensis* infection

**Figure 5:**
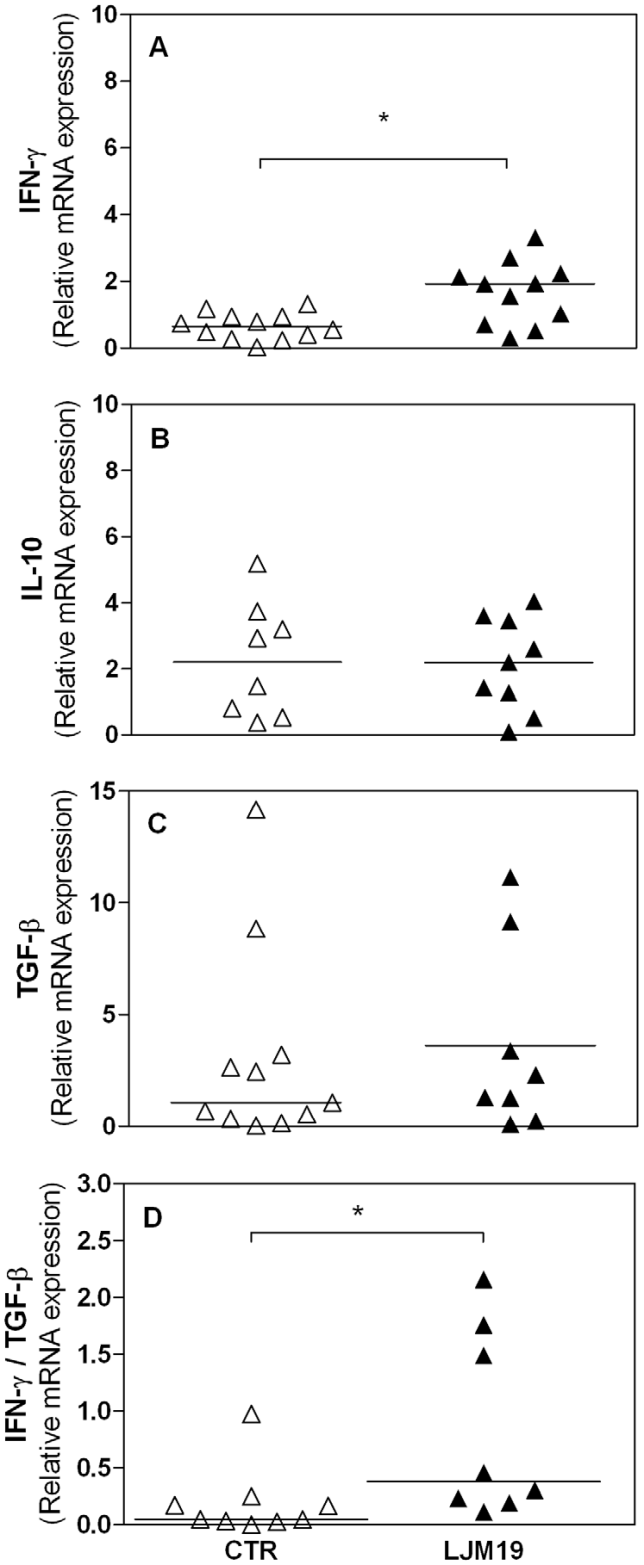
Cytokines production by lymph node cells from hamsters immunized with LJM19 DNA plasmid in *L. braziliensis* infection. Hamsters were inoculated three times in the right ear with DNA plasmid LJM19 salivary protein (closed triangle; 9 hamsters per group total) or empty DNA plasmid (CTR – open triangle; 10 hamsters per group total) and were challenged in the left ear with 10^5^ *L. braziliensis* in the presence of *Lu. intermedia* SGS. The IFN-γ (A), IL-10 (B) and TGF-β (C) relative mRNA expression was evaluated by Real-Time PCR 5 weeks after the infection. The ratio IFN-γ/TGF-β obtained in A and C for each group was compared (D). Points represent each animal, experiments were repeated three times and were evaluated by Mann-Whitney non-parametric t test. *p<0.05.

In order to investigate the mechanism involved in the protection conferred by LJM19 DNA plasmid immunization, the cytokine expression in draining lymph node cells was evaluated 5 weeks after challenge with *L. braziliensis + Lu. intermedia* SGS by Real-Time PCR (Fig. 5). There was a significant increase in the IFN-γ expression in LJM19 DNA plasmid immunized group, p = 0.011 (Fig. 5A). However, no differences were found in IL-10 (Fig. 5B) or TGF-β (Fig. 5C) expression in hamsters immunized with LJM19 DNA plasmid compared to control group. Moreover, the ratio of IFN-γ/TGF-β expression was significantly higher (p = 0.011) in LJM19 DNA plasmid immunized hamsters compared with the control group (Fig. 5D). Together, these results suggest that immunization with a defined protein induces a protective immune response, with higher expression of pro-inflammatory cytokine (IFN-γ).

## Discussion

In this study, we observed that *Lu. longipalpis* SGS or DNA plasmid coding for the salivary protein LJM19 protected hamsters against a challenge composed of *L. braziliensis* plus SGS from either *Lu. longipalpis* or from *Lu. intermedia*. Hamsters are a good model to evaluate protection against *L. braziliensis*, since they are highly susceptible to this infection [16], [17]. It has been shown that exposure to sand fly saliva induces a protective immune response against *Leishmania* [7], [8], [18]. The mechanism of protection conferred by vectors' saliva is not fully understood. However, reports in the literature have shown that the induction of a DTH response by whole saliva or by single salivary protein is a marker of protection [8], [9], [19]. Our results using a DNA plasmid coding LJM19 salivary protein from *Lu. longipalpis* indicate that a cellular immune response could be the mechanism involved in the protection. The immunization with DNA coding this protein does not induce antibody production [9], but a DTH reaction was observed in hamsters immunized and challenged with either *Lu. longipalpis* or with *Lu. intermedia* SGS.

In hamsters immunized with *Lu. longipalpis* SGS, the presence of *Lu. longipalpis* SGS in the inoculum did not lead to a reduction in ear thickness. This result suggests dissociation between parasite load and ear thickness, as already shown in other studies [20], [21]. Surprisingly, we did not observe any exacerbation in the infection caused by *L. braziliensis* plus *Lu. longipalpis* SGS in unimmunized hamsters, as shown by other studies [4], [22]. Since hamsters are highly susceptible to *Leishmania* [16], [17], possibly sand fly saliva did not exacerbate the infection. The dissociation between parasite load and lesion development has already been demonstrated [20], [21]. In fact, the ear thickness observed in the hamsters challenged with *L. braziliensis* plus *Lu. longipalpis* SGS could be attributed to differences in the inflammatory infiltrate, characterized by an intense cellular recruitment to the site of infection, as observed in the DTH reaction. On the other hand, the challenge with *Lu. intermedia* SGS in hamsters immunized with *Lu. longipalpis* induced a less pronounced cell recruitment, but still able to control the infection, as observed by the reduced ear thickness. In this way, the intensity of cell recruitment induced by SGS in the inoculum seems to be a key factor to detect lesion development. Using mice infected with *L. amazonensis*, another model of high susceptibility infection, immunization with *Lu. longipalpis* SGS also protected mice against *L. amazonensis* infection in the absence or presence of saliva [23]. On the other hand, BALB/c mice immunized with *Lu. intermedia* SGS and challenged with *L. braziliensis* plus *Lu. intermedia* SGS showed an exacerbation of *L. braziliensis* infection [5]. The possible mechanism underlying these different outcomes is the cytokine profile induced in each case. In *Lu. longipalpis* SGS immunized mice, there was an induction of Th1-type cytokines [23] whereas in *Lu. intermedia* SGS immunized mice, a Th2 profile was observed [5]. Besides that, *Lu. intermedia* immunization did not induce cellular immune response, but did generate antibody production and a mixed cellular (Th1–Th2) response, which favored parasite establishment and survival [5]. It is important to consider that the differences observed in these papers are not possibly due only to different sand flies, but also to the amount of Leishmania promastigotes as well as the parasite species used in the inoculum. Another aspect to consider is the type of challenge employed: natural transmission using infected sand flies or only *Leishmania* injected by needle. Recently, Peters *et al* [19] demonstrated that the use of natural transmission of *Leishmania* abrogates the immunity conferred by killed parasite vaccine, which was shown to be protective against a challenge by needle with *L. major* only. Although we used a challenge by needle, our infection was composed by *L. braziliensis* with saliva, which is important in the outcome of infection. Our attempt is to induce an immune response against saliva present at the moment of infection which may collaborate to create an inhospitable environment for the establishment of *Leishmania* infection that may involve direct killing of parasite. Sand fly saliva alters the local microenvironment by modulating the cytokine profiles and the co-stimulatory molecules expression [24]. Therefore, the immune response against salivary components and its presence in the challenge possibly favors the host in the early events after the infection.

Immunization with *Lu. longipalpis* SGS decreased the expression of IL-10 and TGF-β in draining lymph node cells of hamsters challenged with *L. braziliensis* + *Lu. intermedia* SGS. Although no differences were observed in IFN-γ expression, the decrease of inhibitory cytokines can contribute to the protective effect during the infection [25]. Surprisingly, the immunization with *Lu. longipalpis* SGS did not change the levels of anti-inflammatory cytokines in the hamsters challenged with parasites plus *Lu. longipalpis* SGS, although the animals showed a reduced parasite load in the ears and lymph nodes. Possibly, in hamsters immunized with whole saliva there were different levels and types of immune response induced by distinct proteins present in the saliva, which could mask some difference in cytokines expression, such as IFN-γ/TGF-β ratio.

Recently, our group demonstrated that immunization with DNA plasmid coding LJM19 protected hamsters against a fatal infection by *L. chagasi* plus *Lu. longipalpis* SGS [9]. In the present study, we investigated whether immunization with this DNA plasmid also protects against *L. braziliensis* plus *Lu. intermedia* SGS. Interstingly, the protection conferred by immunization with LJM19 DNA plasmid is very similar to that obtained with total *Lu. longipalpis* SGS. The immunization with LJM19 lead to a pronounced Th1 response with an increase in IFN-γ expression and no differences in IL-10 or TGF-β were noticed. Therefore, the ratio IFN-γ/TGF-β expression was higher than controls. The same cytokine response was observed in protected hamsters immunized with LJM19 and challenged with *L. chagasi* plus *Lu. longipalpis* SGS [9].

The results showed in this study suggest cross-reactivity between *Lu. longipalpis* and *Lu. intermedia* SGS. De Moura et al (2007) showed that SDS-PAGE profiles from *Lu. intermedia* and *Lu. longipalpis* SGS displayed bands migrating at similar molecular weight. However, *Lu. intermedia* immune sera from BALB/c mice did not recognize any proteins present in *Lu. longipalpis* SGS, with the exception of the ∼45 kDa one [5]. Although few studies in the literature have shown the direct effect of *Lu. longipalpis* saliva on innate immune response cells [26], [27], we can also speculate that some of these mechanisms, such as gamma/delta+ T cells in skin or parasite-saliva sharing antigens in their glycan moiety, could also contribute to the protection conferred by the immunization with *Lu. longipalpis* SGS. This has already been suggested in another model where immunization with *Lu. longipalpis* SGS was able to protect mice against *L. amazonensis* without saliva [23]. However, it is intriguing that LJM19 also protected hamsters in a similar way. Further studies are necessary to clarify whether LJM19 and *Lu. intermedia* proteins share common motifs and whether DTH induced by immunization with LJM19 is a consequence of this cross reactivity.

In conclusion, our results confirm the immunogenic ability of *Lu. longipalpis* saliva and a defined salivary protein, LJM19, in hamsters and, for the first time, we show the protective potential of this compounds against *L. braziliensis* infection plus *Lu. intermedia* SGS. These findings suggest that LJM19 could be applied as a component vaccine candidate in different models or even as adjuvant against other pathogens.

## Supporting Information

Figure S1 **Production of antibody against saliva in serum samples from hamsters immunized with** ***L. longipalpis*** **SGS or LJM19 DNA plasmid.** Hamsters were inoculated three times in the right ear with *Lu. longipalpis* SGS (closed squares) or LJM19 DNA plasmid (closed triangles) or with saline (open squares). After 48 hours, the serum samples were collected and the total IgG against *L. longipalpis* SGS was measured by ELISA. Points represent each animal, experiments were repeated three times and were evaluated by Kruskal-Wallis non-parametric test. **p<0.01; ***p<0.001.
(TIF)

Figure S2 **Cytokine expression in lymph node cells from hamsters immunized with** ***Lu. longipalpis*** **followed by** ***L. braziliensis*** **infection.** Hamsters were inoculated three times in the right ear with *Lu. longipalpis* SGS (closed symbols) or with saline (open symbols) and were challenged in the left ear with 10^5^ *L. braziliensis* in the presence of *Lu. intermedia* (squares) or *Lu. longipalpis* SGS (circles). IFN- γ, IL-10 and TGF-β relative mRNA expression was evaluated by Real-Time PCR at three (left column) and eight (right column) weeks after the infection. Points represent each animal, experiments were repeated three times and were evaluated by Mann-Whitney non-parametric t test. *p<0.05.
(TIF)

Figure S3 **Parasite load in LJM19 immunized hamsters following infection with** ***L. braziliensis*** **.** Hamsters (12 per group total) were inoculated three times in the right ear with DNA plasmid coding LJM19 salivary protein or empty DNA plasmid (CTR) and were challenged intradermally in the left ear with 10^5^ *L. braziliensis* stationary promastigotes in the presence of *Lu. intermedia* SGS. Five weeks after the infection, the parasite load was evaluated in the draining lymph node by LDA and estimated by ELIDA. Experiments were repeated three times and were evaluated by Mann-Whitney non-parametric t test. *p<0.05.
(TIF)

Figure S4 **Parasite load in LJM19 DNA plasmid or** ***Lu. longipalpis*** **SGS immunized hamsters following infection with** ***L. braziliensis*** **.** Hamsters (9 per group total) were inoculated three times in the right ear with DNA plasmid coding LJM19 salivary protein or *Lu. longipalpis* SGS (closed bars) or empty DNA plasmid (CTR) or Saline (open bars) and were challenged intradermally in the left ear with 10^5^ *L. braziliensis* stationary promastigotes in the presence of *L. intermedia* SGS. The parasite load was evaluated 5 weeks after infection in the ear (A) and draining lymph node (B) by LDA, estimated by ELIDA. Bars represent the median and standard errors of the means. Experiments were repeated three times and were evaluated by ANOVA (Kruskal-Wallis) analysis with Dunn's post-test. *p<0.05; **p<0.01.
(TIF)
