# Supplementary figures and images for: Lutzomyia longipalpis Saliva or Salivary Protein LJM19 Protects against Leishmania braziliensis and the Saliva of Its Vector, Lutzomyia intermedia
Source: PLoS Negl Trop Dis. 2011 May 31;5(5):e1169. doi: 10.1371/journal.pntd.0001169 (PMC3104964; doi:10.1371/journal.pntd.0001169)

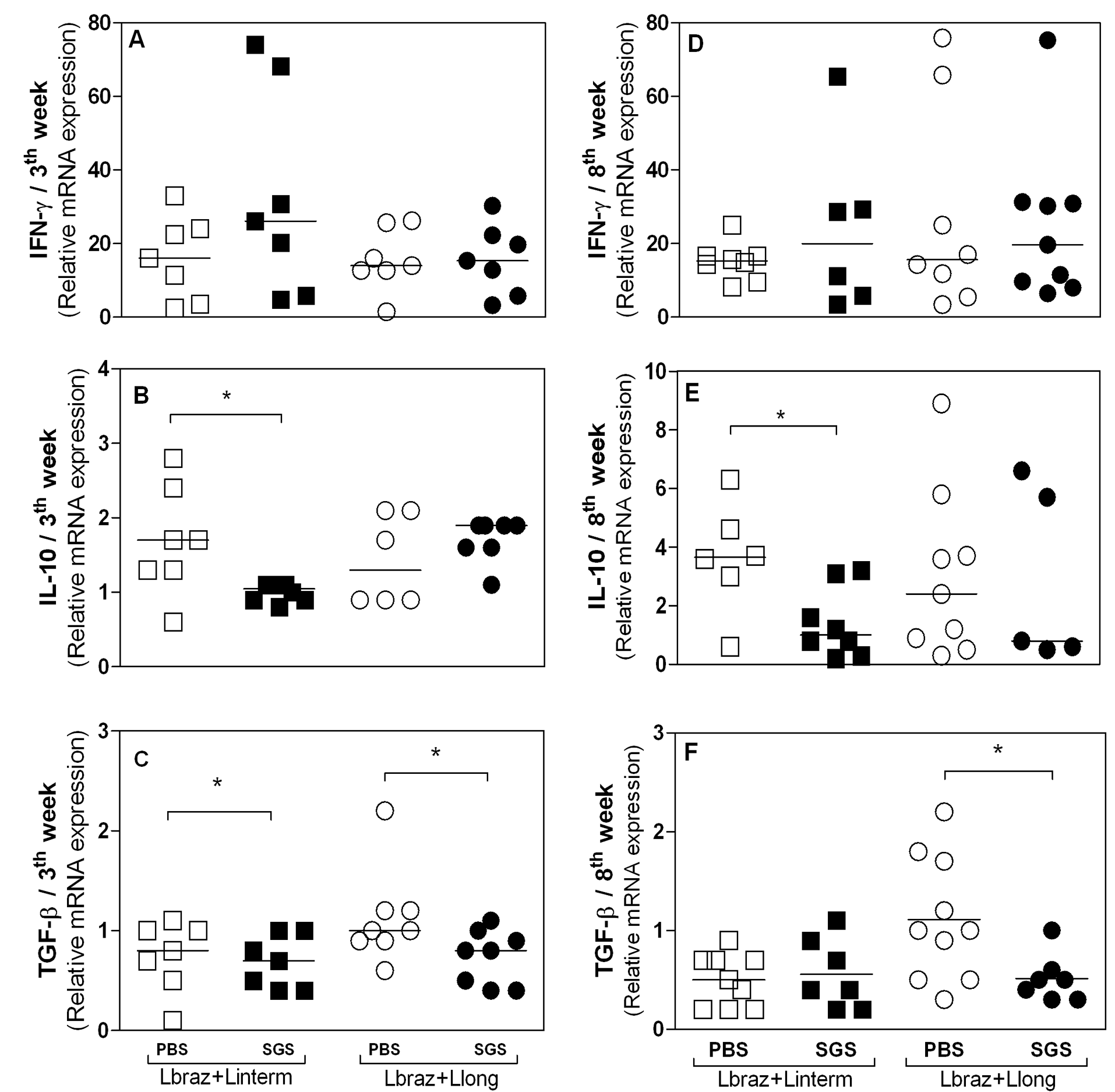

Supplement: Figure S2 — Cytokine expression in lymph node cells from hamsters immunized with Lu. longipalpis followed by L. braziliensis infection. Hamsters were inoculated three times in the right ear with Lu. longipalpis SGS (closed symbols) or with saline (open symbols) and were challenged in the left ear with 105 L. braziliensis in the presence of Lu. intermedia (squares) or Lu. longipalpis SGS (circles). IFN- γ, IL-10 and TGF-β relative mRNA expression was evaluated by Real-Time PCR at three (left column) and eight (right column) weeks after the infection. Points represent each animal, experiments were repeated three times and were evaluated by Mann-Whitney non-parametric t test. *p<0.05. (TIF) [file pntd.0001169.s002.tif]

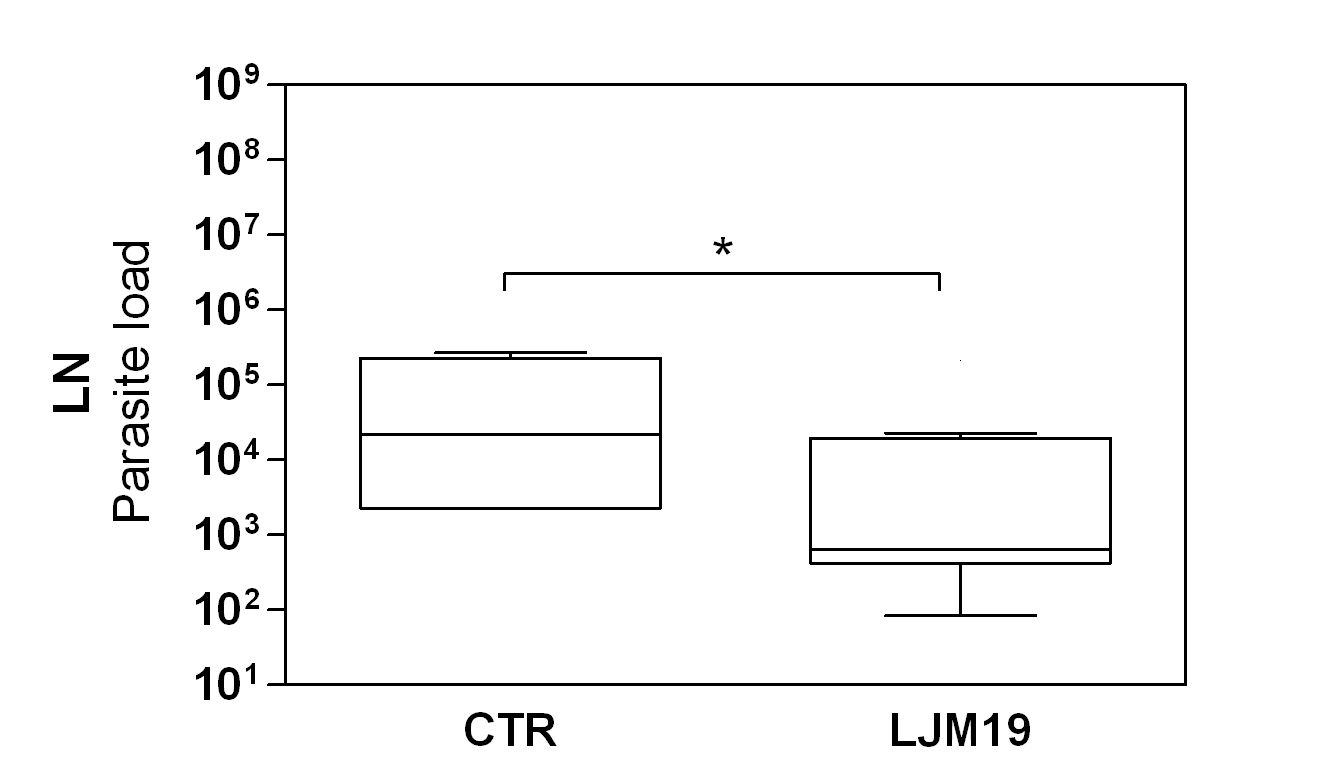

Supplement: Figure S3 — Parasite load in LJM19 immunized hamsters following infection with L. braziliensis . Hamsters (12 per group total) were inoculated three times in the right ear with DNA plasmid coding LJM19 salivary protein or empty DNA plasmid (CTR) and were challenged intradermally in the left ear with 105 L. braziliensis stationary promastigotes in the presence of Lu. intermedia SGS. Five weeks after the infection, the parasite load was evaluated in the draining lymph node by LDA and estimated by ELIDA. Experiments were repeated three times and were evaluated by Mann-Whitney non-parametric t test. *p<0.05. (TIF) [file pntd.0001169.s003.tif]

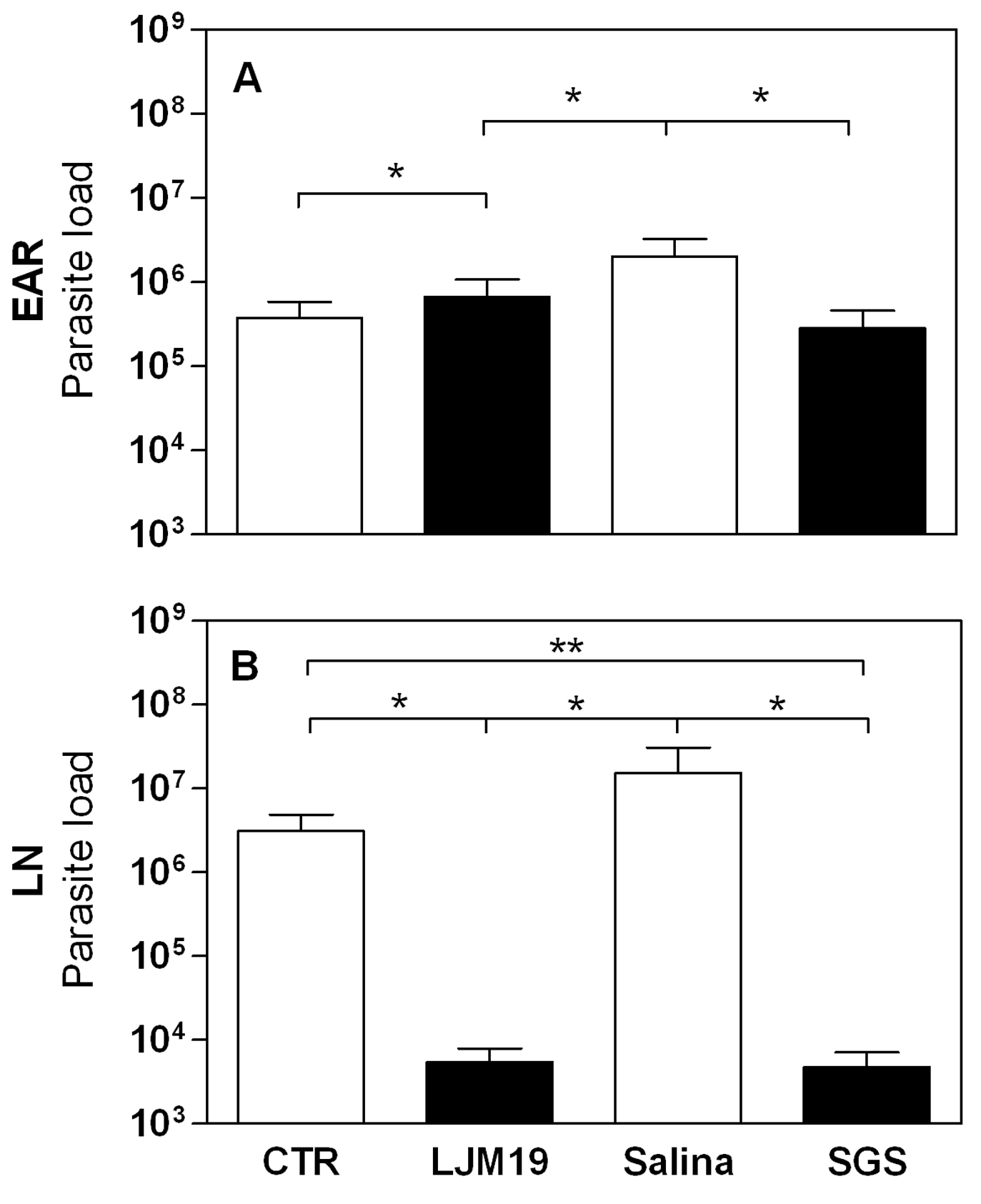

Supplement: Figure S4 — Parasite load in LJM19 DNA plasmid or Lu. longipalpis SGS immunized hamsters following infection with L. braziliensis . Hamsters (9 per group total) were inoculated three times in the right ear with DNA plasmid coding LJM19 salivary protein or Lu. longipalpis SGS (closed bars) or empty DNA plasmid (CTR) or Saline (open bars) and were challenged intradermally in the left ear with 105 L. braziliensis stationary promastigotes in the presence of L. intermedia SGS. The parasite load was evaluated 5 weeks after infection in the ear (A) and draining lymph node (B) by LDA, estimated by ELIDA. Bars represent the median and standard errors of the means. Experiments were repeated three times and were evaluated by ANOVA (Kruskal-Wallis) analysis with Dunn's post-test. *p<0.05; **p<0.01. (TIF) [file pntd.0001169.s004.tif]
